# Brain volumes in congenital heart disease from childhood to adulthood: A pooled neuroimaging study

**DOI:** 10.1016/j.ynirp.2026.100379

**Published:** 2026-07-08

**Authors:** Asuka Toyofuku, Nadja Naef, Valentin Rousson, Melanie Ehrler, Flavia M. Wehrle, Ladina Schlosser, Michael von Rhein, Rabia Liamlahi, Matthias Greutmann, Oliver Kretschmar, Beatrice Latal, Ruth Tuura O'Gorman

**Affiliations:** a Child Development Center, University Children's Hospital Zürich, Zürich, CH-8008, Switzerland; b Division of Biostatistics, Center for Primary Care and Public Health (Unisanté), University of Lausanne, Switzerland; c Children's Research Center, University Children's Hospital Zürich, Zürich, CH-8008, Switzerland; d Department of Cardiology, University Heart Center, University Hospital Zurich, Zürich, CH-8008, Switzerland; e Pediatric Cardiology, Pediatric Heart Center, University Children's Hospital Zürich, Zürich, CH-8008, Switzerland; f MR Research Center, University Children's Hospital Zürich, Zürich, CH-8008, Switzerland

**Keywords:** Congenital heart disease, Brain volume, MRI, Trajectory, Children, Adolescents

## Abstract

Complex congenital heart disease (CHD) is associated with reduced brain volumes, but little is known about the brain developmental trajectory in CHD beyond childhood, which is a critical period for brain maturation. This study reports alterations in brain volumes from a large cross-sectional dataset of patients with CHD and controls, with an age range from childhood to young adulthood.

Patients and controls underwent 3 T cerebral MRI and overlapping cognitive assessments. Images were processed using Freesurfer 5.3. The dataset comprised 311 participants, 128 CHD and 183 controls aged between 9 and 32 years (male: 52.1%). Associations between the total brain and grey matter, white matter, and cerebrospinal fluid (CSF) volumes and age, sex, group (CHD vs. controls) and maternal education were analysed using linear mixed models.

Global and total grey/white matter volumes were smaller in patients with CHD compared to controls (*p* < 0.001), whereas CSF volumes did not differ significantly between groups (*p* = 0.23). No significant interaction between the group, sex and age was found. Larger global brain volume was associated with higher maternal education (*p* < 0.001) and higher IQ (*p* < 0.001).

Consistent lower brain volume in CHD than in controls throughout childhood and young adulthood suggests that there is no convergence towards healthy peers in CHD brain volumes over time. Functional correlates of smaller brain volumes underscore the importance of longitudinal studies in better understanding the evolution and determinants of impaired brain development in CHD populations.

## Highlights

• Brain volume reductions in CHD across development are underexplored.
• Brain volume changes in CHD patients and controls aged 9-32 years were explored.
• CHD patients show consistently smaller brain volumes from childhood to adulthood.
• There is no evidence for a catch-up brain growth over time for CHD patients.

## 1 Introduction

Congenital heart disease (CHD) is one of the most common congenital malformations (Jenkins et al., 2019). With more than 90% of children with complex CHD expected to reach adulthood, CHD is now considered a life-long condition (Jenkins et al., 2019; Marelli et al., 2016). Despite enhanced survival rates attributed to therapeutic improvements, the CHD population face a higher risk of neurodevelopmental impairments (Marino et al., 2012; Kovacs and Bellinger, 2021), which are related to various brain structural changes, including reduced brain volumes (Bonthrone et al., 2021).

Several longitudinal neuroimaging studies have investigated the trajectory of brain growth in CHD from the foetal to the neonatal period, indicating that neuroanatomic alterations begin prenatally and that CHD patients exhibit different brain growth patterns compared to control infants (O'Brien et al., 2026; Rollins et al., 2021; Ortinau et al., 2018; Cromb et al., 2023; Peyvandi et al., 2018; Claessens et al., 2019). One longitudinal analysis of total brain growth trajectories from 32 weeks of gestation to 3 months postnatally found a flatter weekly growth slope in patients with CHD compared with healthy controls, suggesting ongoing deviations from the normal trajectory beyond the foetal period (Ortinau et al., 2018). Another longitudinal study assessed pre- and post-operative brain growth trajectories in infants with CHD compared to healthy infants, and the results emphasised the heterogeneous trajectories among CHD, ranging from greatly impaired to stable or even accelerated growth (Cromb et al., 2023).

This variability in brain growth in CHD can be related to the heterogeneity of the pathophysiology and severity of CHD. For example, one study found slower brain growth in the hypoplastic left heart syndrome (HLHS)/transposition of the great arteries (TGA) group but not in other CHD types between 18 and 40 weeks of gestation (Rollins et al., 2021). Neonates with HLHS also showed slower perioperative brain growth compared to TGA, possibly related to the differences in cardiac physiology (Peyvandi et al., 2018). In addition, the complex CHD populations are at risk for severe brain injury (Owen et al., 2011), which further impairs perioperative brain growth in CHD neonates, resulting in slower growth rates (Peyvandi et al., 2018; Claessens et al., 2019). However, another longitudinal study found similar brain growth in both HLHS and TGA children between 3 and 9 years of age (Hiraiwa et al., 2020), suggesting that the rate of brain growth in the CHD population may be dependent on the examined age period.

While these longitudinal studies of brain growth in CHD only assessed a limited period, mainly from the foetal to the neonatal period or in early childhood, other studies have examined differences in brain volumes in cross-sectional studies of children, adolescents or young adults (Bolduc et al., 2018), demonstrating that some volumetric deficits persist into adulthood. However, these cross-sectional studies examined patients at a particular developmental stage or within a narrow age range, and the pattern of brain growth and development over time beyond the neonatal period remains mostly unknown. An understanding of the brain developmental curves for children and adolescents with CHD is crucial, given that adolescence is a period of extensive brain maturation (Gilmore et al., 2018). For example, the regionally-specific reduction of grey matter volume seen during adolescence (forming an inverted U-curve with its peak before adolescence) might reflect pruning in use-dependent selective synapse elimination (Shaw et al., 2008). In contrast, white matter volume is reported to increase linearly throughout late childhood and adolescence, with an increasing number of oligodendrocytes responsible for producing myelin. While many regions are myelinated in the early years of life, mature myelination patterns in the neocortex are attained only in adulthood (Gilmore et al., 2018; Lebel et al., 2012; Tamnes et al., 2010). An understanding of these brain development and maturation patterns reported beyond childhood is currently lacking in the CHD population.

Therefore, the aim of this study is to investigate developmental changes in brain volumes in a cross-sectional sample of patients with CHD, covering a broad age range between 9 and 32 years of age. Although only longitudinal studies can track brain development from childhood to adulthood, maintaining a large sample size over such a long period is challenging; thus, we decided to pool multiple cohorts and examine age-related effects across childhood and adulthood using statistical modelling. We have mainly focused on the global structures in this paper, as prior work has most consistently demonstrated global reductions, with region-specific differences often attenuated after accounting for overall brain size (Latal et al., 2016; Naef et al., 2023a). Based on the brain volumetric reductions reported in the cross-sectional neuroimaging studies on adolescents and young adults (Bolduc et al., 2018; Aleksonis and King, 2022), we hypothesise that total brain volumes of CHD remain consistently lower at any age than those of healthy counterparts. We also expect that grey matter shows a decrease with age, while white matter shows a linear increase.

## 2 Materials and methods

### 2.1 Participants

Data from four different cross-sectional studies were combined (the flowchart can be found in Supplementary Fig. 1). Patients with CHD were recruited from the University Children's Hospital Zurich or the outpatient clinic for adults with CHD at the University Hospital Zurich. Inclusion criteria for the pooled analysis were: having a CHD that required a cardiopulmonary bypass surgery before six years of age and having undergone cerebral magnetic resonance imaging, including a high-resolution 3D T1-weighted volumetric scan. Exclusion criteria were: having a diagnosis of a genetic disorder, a severe neurological comorbidity, or not being fluent in the German language.

In the *brain function in congenital heart disease (BFCHD)* study (Naef et al., 2023b), children with different types of CHD who underwent cardiopulmonary bypass surgery before the age of 6 and healthy controls aged 9 to 11 years of age were recruited. In this study, children underwent neurocognitive assessment and brain imaging at the University Children's Hospital between July 2016 and July 2019. The *Teenheart* study examined adolescents with CHD and healthy controls aged 10 to 15 years (Ehrler et al., 2019). Participants completed neurocognitive assessment and brain imaging between April 2019 and September 2021. The *LEHOPS* study (von Rhein et al., 2011) included adolescents with different types of CHD aged 11.4 to 16.9 years who underwent cardiopulmonary bypass surgery between 1995 and 1998 at the University Hospital Zurich. In parallel, healthy controls were recruited for this study or participated in the previous studies. The *Grown-up CHD (GUCH)* study recruited adults with CHD who attended the outpatient clinic for adults with CHD at the University Hospital Zurich aged 18 to 32. For comparison, healthy controls were recruited, who were matched for age and sex (Schlosser et al., 2022). There were 14 participants who joined both the *BFCHD* study (2016-2019) and the *Teenheart* study (2019-2021). For these participants, we retained the earlier (*BFCHD*) time point in the main analysis to preserve a more balanced age distribution, given the small number of younger participants (<11 years).

All procedures performed in the included studies were in compliance with the Declaration of Helsinki, relevant national laws, and institutional guidelines, and were approved by the Cantonal Ethics Committee of Zurich. Specifically, approval was obtained for the *BFCHD* study on 16.02.2016 (Reference Number: PB_2016-00496), for the *Teenheart* study on 12.02.2019 (Reference Number: PB_2019–00035), for the *LEHOPS* study on 03.02.2009 (Reference Number: KEK StV 23/04), and for the *GUCH* study on 07.04.2016 (Reference Number: PB_2016-01348). Written informed consent was obtained from all individual participants or their legal guardians.

### 2.2 Measurements

In children and adolescents, the intelligence quotient (IQ) was assessed using the Wechsler Intelligence Scale for Children – Fourth Edition (WISC-IV) (Petermann, 2012). In adults, the Wechsler Adult Intelligence Scale, Fourth Edition, WAIS-IV (Daseking et al., 2014) was used. IQ was measured with a full-form IQ for the *GUCH* study or a short-form using four subsets for the other three studies (Waldmann, 2008). The WISC-IV short form was corrected based on a previously established correction formula for children and adolescents (Waldmann, 2008; Ehrler et al., 2020). Demographic variables were collected through questionnaires or obtained from chart review. Maternal education was measured using a 6-point scale (1 = no high school degree, 2 = high school degree, 3 = apprenticeship, 4 = higher diploma for craftsmen or craftswomen, 5 = advanced diploma of higher education, 6 = university degree). Medical variables were collected from the medical records and included: CHD diagnosis, number of cardiopulmonary bypass surgeries and age at first cardiopulmonary bypass surgery.

### 2.3 MRI acquisition and processing

Cerebral magnetic resonance imaging was performed at the University Children's Hospital Zurich on a 3 T GE MR750 (*BFCHD, Teenheart, GUCH*) scanner or a 3 T GE HD. xt scanner (*LEHOPS*). Three-dimensional anatomic images of the entire brain were obtained using a T1-weighted spoiled gradient echo pulse sequence (SPGR) to assess brain volumes. SPGR images were acquired using the following scan parameters: *BFCHD, Teenheart, & GUCH*: TR/TE = 11/5 ms; inversion time = 600 ms; flip angle = 8°; reconstructed matrix = 256×256; FOV = 26 cm; 176 contiguous axial slices, 1 mm slice thickness. *LEHOPS:* TR/TE 25/5 m, inversion time = 450 ms, flip angle = 13°, FOV = 24 cm, acquisition matrix = 352 × 224, slice thickness 1.2 mm, image resolution 0.47 × 0.47 mm.

Cortical reconstruction and volumetric segmentation were performed with the FreeSurfer image analysis suite version 5.3, using the aparc. a2009s + aseg.mgz file (representing the Destrieux atlas). This is freely available online (http://surfer.nmr.mgh.harvard.edu/). It is reported that Freesurfer provides robust and reliable measurements across different scanners and field strengths (Han et al., 2006; Jovicich et al., 2009). Version 5.3 was used to maintain a consistent analysis pipeline across all datasets, since the earlier datasets (*LEHOPS*) had been segmented in that version, which was the current one at the time. However, it is known that different Freesurfer versions can yield different outcomes (Bigler et al., 2020; Filip et al., 2022); therefore, as a supplementary analysis, TBV was compared between versions 5.3 and 7.1 in our most recent dataset (*Teenheart* study). For statistical analyses, we used standard Freesurfer outputs, in which volumes were pooled across both hemispheres within the pipeline. A visual inspection was conducted to check the quality of the FreeSurfer segmentation. Of note, no manual corrections were made, but scans with poor image quality or poor segmentation quality were excluded (Mayer et al., 2016). Images were regarded as poor quality if there were clear artefacts on several slices, and/or if there were several missed or over-segmentations throughout the image. Common brain lesions in patients with CHD have been previously described (von Rhein et al., 2011). Segmentation was successful with 311 participants. Brain volumes analysed included: total brain volume (TBV), total grey matter (GM), total white matter (WM) and cerebrospinal fluid (CSF). Total GM volume includes both cortical and subcortical GM. The CSF volume was derived from FreeSurfer outputs by combining the volume of the “CSF” label in aseg. stats (which predominantly reflects non-ventricular CSF, mainly subarachnoid and sulcal CSF) with ventricular volumes (including lateral ventricles, inferior lateral ventricles, choroid plexus, 3rd, 4th ventricles and Cavum Septi Pellucidi, which was labelled as 5th ventricle in the Freesurfer output).

### 2.4 Statistical analysis

Descriptive statistics included mean and standard deviation (SD) for continuous variables and counts and proportions for categorical variables. Groups (such as CHD vs. controls, CHD with univentricular vs. biventricular defects or cyanotic vs. acyanotic) were compared using a *t*-test for continuous variables or a Chi-squared test for categorical variables. Associations between continuous variables (such as brain volume vs. IQ) were assessed via Spearman's rho correlation. The primary demographics are similarly compared between the final sample and the removed sample.

In our main analysis, associations between the different brain volumes (TBV, WM, GM and CSF) and age, sex, group (CHD vs. controls) and maternal education were analysed using linear mixed models. CSF volumes were log-transformed in order to approximate normality. Age and maternal education were standardised to have a SD of one. A random study effect has been included in all the models to take into account the possible dependencies among the participants from the same study.

All two-way interactions between age, sex and group have also been tested for all outcomes. This was done by testing for the significance of the coefficients related to terms age∗sex, age∗group and group∗sex, which were included as additional covariates in the model. As a sensitivity analysis, the analysis was repeated by including only patients with a biventricular defect, only cyanotic patients, or only acyanotic patients. This could not be done for univentricular patients since their number was limited (N = 14). For a subgroup analysis, a similar analysis was conducted to compare clinical subgroups of CHD patients (univentricular vs. biventricular defects, acyanotic vs. cyanotic, two or more vs one surgery). All models were fitted using the lmer routine available in the lme 4 library from the R software (R core team, version 4.3.1, URL http://Rproject.org/). Two-tailed *p*-values <0.05 were considered significant. To give an idea of the clinical significance of our results, the coefficients estimated in our models were divided by the residual SD, to be interpretable as standardized mean differences (or Cohen's delta), either between the two groups defined by a binary predictor (sex, group), or for an increase of one SD regarding a quantitative predictor (age, maternal education). Values of delta = 0.2, 0.5 and 0.8 are usually regarded as small, medium and large effects, respectively.

## 3 Results

### 3.1 Sample description

In total, 422 participants were initially reviewed in this study (patients *N* = 216). Among those, 111 participants were excluded due to a lack of or poor quality of T1 images, age restrictions, or overlap between the studies. The final sample consisted of 311 (128 patients and 183 controls). The final sample was not different from the excluded subjects in terms of sex proportion (male: 52.1% vs 50.6%, *p* = 0.91), IQ (106 vs 102, *p* = 0.06) and maternal education (4.2 vs 4.0, *p* = 0.31), but significantly older than the excluded sample (16.12 vs 13.82, *p* < 0.001). There were significant differences in the proportion of cyanotic CHD (57.8% vs 75.0%, *p* = 0.04) and univentricular CHD (10.9% vs 26.8%, *p* = 0.007) between the final and excluded sample. A flowchart depicting inclusion and exclusion numbers throughout the different studies can be found in Supplemental Fig. 1.

Demographics and cardiac variables of the final sample are listed in Table 1, stratified into four study cohort categories. Comparisons of the demographics among CHD patients are shown in Supplemental Table 1. The detailed CHD diagnosis is shown in Supplemental Table 2. Overall, the proportion of males was 59% for patients with CHD and 48% for controls (*p* = 0.07), and there was no significant age difference (16.6 vs 15.4, *p* = 0.06). The controls had higher maternal education (4.5 vs 3.8, *p* < 0.001) and higher IQ (109 vs 101, *p* < 0.001) than the CHD group. IQ was negatively associated with age (rho = −0.16, p = 0.006), positively associated with maternal education (rho = 0.29, *p* < 0.001), TBV (rho = 0.28, *p* < 0.001), WM (rho = 0.19, *p* = 0.001) and GM (rho = 0.32, *p* < 0.001), but not associated with CSF (rho = −0.05, *p* = 0.43). All our correlations have been calculated over the whole sample, including patients with CHD and controls.

**Table 1:** Characteristics of CHD and controls stratified by study cohort.

	BFCHD study		Teenheart study		LEHOP study		GUCH study		Complete sample	
	CHD N = 16	Controls N = 14	CHD N = 46	Controls N = 84	CHD N = 45	Controls N = 32	CHD N = 21	Controls N = 53	CHD N = 128	Controls N = 183
Male, N (%)	11 (68.8)	8 (57.1)	26 (56.5)	40 (47.6)	21 (46.7)	12 (37.5)	17 (81.0)	27 (50.9)	75 (58.6)	87 (47.5)
Age (year), Mean (SD)	10.25 (0.45)	9.93 (0.83)	13.55 (1.16)	12.95 (1.39)	13.76 (1.53)	13.91 (1.43)	26.69 (3.76)	25.90 (13.30)	15.37 (5.49)	16.64 (6.36)
Maternal education, Mean (SD)	3.94 (1.18)	4.42 (1.08)	3.89 (1.30)	4.76 (1.10)	3.62 (1.17)	4.10 (0.92)	3.85 (1.04)	4.27 (0.82)	3.80 (1.20)	4.48 (1.03)
IQ∗, Mean (SD)	98.36 (12.82)	111.86 (8.38)	100.67 (12.08)	110.78 (9.43)	105.58 (13.84)	111.00 (9.58)	94.15 (10.47)	104.02 (12.15)	101.09 (13.08)	108.87 (10.69)
Univentricular CHD, N (%)	2 (12.5)		8 (17.4)		2 (4.4)		2 (9.5)		14 (10.9)	
Cyanotic CHD, N (%)	13 (81.2)		29 (63.0)		21 (46.7)		11 (52.4)		74 (57.8)	
Age at the 1st bypass surgery, months Median [IQR]	0.52 [0.24, 2.14]		1.50 [0.27, 4.87]		10.44 [5.28, 27.60]		7.32 [2.16, 16.56]		4.40 [0.41, 10.21]	
One bypass surgery, N (%)	13 (81.2)		33 (71.7)		37 (82.2)		12 (57.1)		95 (74.2)	

Mean (SD), N (%), Median [IQR], IQ: Intelligence quotient, CHD: congenital heart disease ∗For GUCH study, a full form IQ was used and for the rest (BFCHD, Teenheart, LEHOP study), a corrected short-form IQ was used.

As a supplemental analysis, we compared TBV estimates derived from FreeSurfer versions 5.3 and 7.1 in our most recent dataset (*Teenheart* cohort) and found that, despite an approximate 5% offset in absolute values, the measures were extremely highly correlated (rho = 0.994, *p* < 0.001).

### 3.2 Brain volume between 9 and 32 years of age

**Fig. 1:**
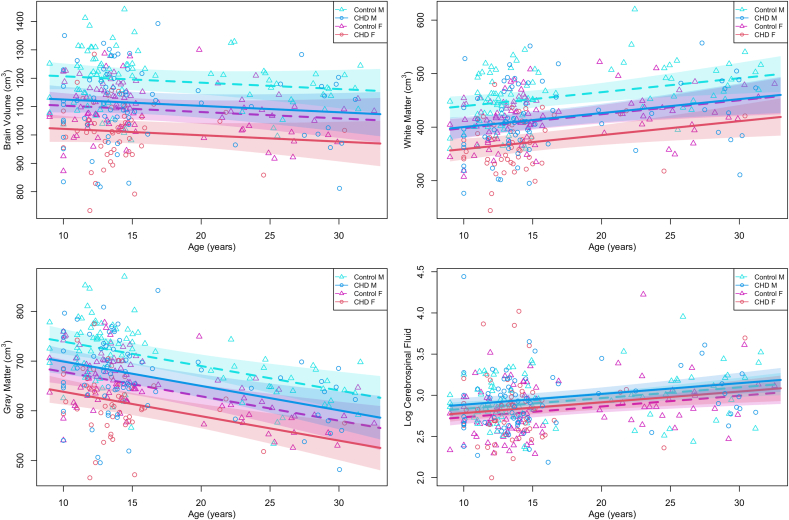
Brain volumes from childhood to young adulthood This figure depicts brain volumes (TBV, WM, GM, and log CSF) plotted against age for males and females in both the CHD and control groups, along with estimated fits from linear mixed models (lines). Statistical details such as standardised mean differences (Cohen's delta), 95% confidence intervals and p-values related to the four predictors in the models (CHD group, male sex, age and SES) are provided in Table 3.

Brain volumetric measures of patients with CHD and controls are presented in Table 2, whereas the fitted models for our four outcomes (TBV, WM, GM and log CSF) are shown in Fig. 1, and the estimated effects (delta) relative to the predictors group, sex, age and maternal education, 95% confidence intervals and p-values are given in Table 3. Compared with controls, patients with CHD had significantly lower TBV (delta = −0.82, p < 0.001), WM (delta = −0.81, p < 0.001), and GM (delta = −0.71, p < 0.001), representing large effects, whereas there was no significant difference in CSF. Association with age was significantly negative for GM (delta = −0.52, p < 0.001), significantly positive, although with a smaller effect for WM (delta = 0.33, p = 0.004) and CSF (delta = 0.24, p < 0.001) and not significant for TBV. Sex was significant for all four outcomes, with males demonstrating consistently higher volumes than females, with some huge effects (e.g. delta = 1.04 for TBV, delta = 1.08 for GM). No significant interaction between the factors of age, sex and group was found. There was thus no statistical evidence that the group effect was different for males and females, and there was also no evidence that brain volumetric differences between CHD and controls improved or worsened across age, since the age∗group interaction was not significant. Higher maternal education was significantly associated with TBV, WM, and GM (although with rather small effects), but not with CSF. Similar results were found in the sensitivity analysis, which are presented in Supplemental Table 3, Supplemental Figs. 2, 3, and 4.

**Table 2:** Brain volumetric measures of CHD and controls stratified by study cohort categories, mean (SD) cm.^3^.

	BFCHD study		Teenheart study		LEHOP study		GUCH study		Complete sample	
	CHD N = 16	Controls N = 14	CHD N = 46	Controls N = 84	CHD N = 45	Controls N = 32	CHD N = 21	Controls N = 53	CHD N = 128	Controls N = 183
TBV	1072.94 (111.50)	1115.20 (119.43)	1041.06 (148.50)	1156.12 (99.34)	1087.05 (111.84)	1168.36 (115.32)	1065.81 (117.32)	1113.04 (97.88)	1065.28 (127.17)	1142.65 (105.05)
WM	374.81 (46.01)	398.98 (57.02)	384.77 (63.59)	432.72 (45.87)	397.68 (56.24)	430.95 (47.88)	428.56 (60.88)	461.75 (52.63)	395.25 (60.34)	438.24 (51.74)
GM	673.28 (67.64)	691.10 (69.44)	631.23 (86.69)	695.62 (58.01)	662.29 (61.26)	705.56 (68.90)	612.03 (61.06)	623.79 (52.58)	644.26 (74.47)	676.21 (67.97)
CSF	20.60 (17.82)	16.98 (4.09)	20.11 (8.35)	18.20 (6.21)	17.50 (7.84)	16.80 (5.63)	22.23 (7.95)	21.86 (9.89)	19.60 (9.82)	18.92 (7.48)

TBV: Total brain volume, WM: Total white matter, GM: Total grey matter, CSF: Cerebrospinal fluid, mean (SD).

**Table 3:** Brain volumetric measures between 9 and 32 years old from the fitted models.

	Effect	delta	95% CI	p-values		Effect	delta	95% CI	p-values
**TBV**	group CHD	−0.82	−1.07; −0.57	<0.001	**WMV**	group CHD	−0.81	−1.06; −0.57	<0.001
	sex male	1.04	0.81; 1.27	<0.001		sex male	0.86	0.63; 1.09	<0.001
	age	−0.14	−0.38; 0.11	0.27		age	0.33	0.11; 0.55	0.004
	maternal education	0.26	0.14; 0.38	<0.001		maternal education	0.24	0.12; 0.36	<0.001
**GMV**	group CHD	−0.71	−0.95; −0.46	<0.001	**CSF**	group CHD	0.15	−0.09; 0.4	0.23
	sex male	1.08	0.84; 1.31	<0.001		sex male	0.30	0.07; 0.53	0.009
	age	−0.52	−0.76; −0.28	<0.001		age	0.24	0.1; 0.37	<0.001
	maternal education	0.26	0.14; 0.38	<0.001		maternal education	0.10	−0.02; 0.22	0.10

TBV: Total brain volume, WM: Total white matter, GM: Total grey matter, CSF: Cerebrospinal fluid, CI = confidence interval.

### 3.3 Subgroup differences in brain volumes

**Fig. 2:**
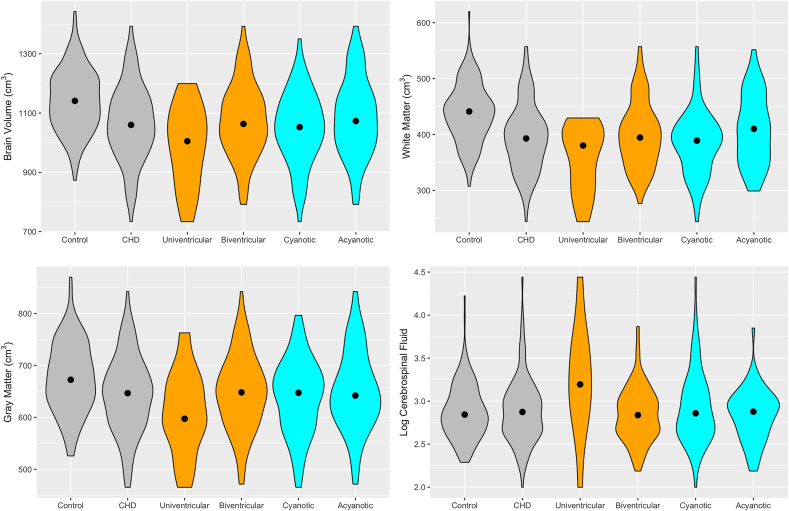
Groupwise difference in total brain, grey matter, white matter, and CSF volumes among the CHD group This figure depicts brain volumes (TBV, WM, GM, and log CSF) plotted by different CHD subgroups and the control group as a reference.

Among patients with CHD, raw comparisons between the univentricular and biventricular groups, between the cyanotic and acyanotic groups, and between patients who underwent one surgery and those who underwent two or more surgeries, for four brain measurements (TBV, WM, GM, CSF volume) are shown in Table 4 and in Fig. 2, the delta values from our adjusted analysis for sex, age and maternal education being presented in Table 5. Those with a univentricular defect had smaller TBV (*p* = 0.01), WM (*p* = 0.009), GM (p = 0.04) and larger CSF (*p* < 0.001) than those with a biventricular defect. For cyanotic or acyanotic types, no significant group difference was found in GM and CSF, but those with cyanotic defect had smaller TBV and WM than those with acyanotic defect (both *p* = 0.04).

**Table 4:** Brain volumetric measures among CHD subgroups, mean (SD) cm.^3^.

	Ventricle type		Cyanotic		Number of surgeries	
	Univentricular N = 14	Biventricular N = 114	Cyanotic N = 74	Acyanotic N = 54	Once N = 95	Two or more N = 33
TBV	989.52 (136.70)	1074.58 (123.40)	1050.49 (122.14)	1085.54 (132.21)	1077.21 (125.01)	1030.92 (128.98)
WM	608.84 (80.89)	648.60 (72.84)	639.72 (72.07)	650.47 (77.89)	652.16 (73.29)	621.50 (74.23)
GM	358.03 (59.89)	399.82 (59.04)	385.73 (57.26)	408.30 (62.51)	398.80 (59.00)	385.02 (63.87)
CSF	30.60 (20.18)	18.25 (6.68)	20.76 (11.73)	18.01 (6.10)	17.72 (6.47)	25.00 (14.81)

TBV: Total brain volume, WM: Total white matter, GM: Total grey matter, CSF: Cerebrospinal fluid, mean (SD).

**Table 5:** Subgroup differences among patients with CHD in brain volumetric measures.

		Effect	delta	95% CI	p-values		Effect	delta	95% CI	p-values
**Univentricualr vs. Biventricular CHD**	**TBV**	group	0.72	0.16; 1.28	0.012	**WMV**	group	0.75	0.19; 1.31	0.009
		sex male	1.00	0.63; 1.38	<0.001		sex male	0.88	0.51; 1.25	<0.001
		age	−0.11	−0.41; 0.19	0.47		age	0.31	0.03; 0.59	0.028
		maternal education	0.36	0.19; 0.54	<0.001		maternal education	0.39	0.22; 0.56	<0.001
	**GMV**	group	0.60	0.03; 1.16	0.039	**CSF**	group	−1.13	−1.69; −0.57	<0.001
		sex male	1.00	0.63; 1.38	<0.001		sex male	0.14	−0.22; 0.51	0.44
		age	−0.44	−0.75; −0.14	0.004		age	0.18	−0.02; 0.38	0.071
		maternal education	0.32	0.14; 0.49	<0.001		maternal education	0.23	0.05; 0.4	0.01
		Effect	delta	95% CI	p-values		Effect	delta	95% CI	p-values
**cyanotic vs. acyanotic CHD**	**TBV**	group	0.38	0.01; 0.76	0.045	**WMV**	group	0.40	0.02; 0.77	0.037
		sex male	1.03	0.66; 1.41	<0.001		sex male	0.91	0.53; 1.28	<0.001
		age	−0.14	−0.44; 0.16	0.35		age	0.27	0; 0.54	0.048
		maternal education	0.37	0.2; 0.54	<0.001		maternal education	0.39	0.22; 0.56	<0.001
	**GMV**	group	0.33	−0.05; 0.7	0.088	**CSF**	group	−0.25	−0.62; 0.12	0.18
		sex male	1.03	0.66; 1.41	<0.001		sex male	0.13	−0.25; 0.5	0.51
		age	−0.47	−0.79; −0.16	0.003		age	0.18	−0.02; 0.38	0.085
		maternal education	0.32	0.15; 0.49	<0.001		maternal education	0.20	0.03; 0.37	0.023
		Effect	delta	95% CI	p-values		Effect	delta	95% CI	p-values
**one surgery vs. 2 or more surgeries**	**TBV**	group	−0.44	−0.85; −0.03	0.037	**WMV**	group	−0.42	−0.83; −0.01	0.047
		sex male	0.98	0.61; 1.35	<0.001		sex male	0.86	0.49; 1.23	<0.001
		age	−0.08	−0.38; 0.23	0.63		age	0.35	0.06; 0.63	0.018
		maternal education	0.38	0.21; 0.55	<0.001		maternal education	0.4	0.23; 0.57	<0.001
	**GMV**	group	−0.4	−0.81; 0.01	0.057	**CSF**	group	0.72	0.31; 1.14	<0.001
		sex male	0.99	0.62; 1.36	<0.001		sex male	0.17	−0.2; 0.54	0.36
		age	−0.42	−0.73; −0.1	0.009		age	0.12	−0.08; 0.32	0.25
		maternal education	0.33	0.16; 0.5	<0.001		maternal education	0.2	0.02; 0.37	0.025

TBV: Total brain volume, WM: Total white matter, GM: Total grey matter, CSF: Cerebrospinal fluid, CI = confidence interval.

Additionally, there was no significant group difference between those with one surgery or those with two or more surgeries in GM, but those with two or more surgeries had smaller TBV (p = 0.04) and WM (p = 0.047) and larger CSF volumes than those with only one surgery (*p* < 0.001).

## 4 Discussion

In this large dataset of patients with CHD and controls between ages 9 to 32 years, patients with CHD had consistently smaller TBV, WM and GM volumes from childhood to young adulthood, but CSF volumes were not different from the controls. The brain size slopes across ages were best depicted using the same mixed linear models for both patients with CHD and controls, such that brain volumes at any age remained lower in the CHD group. This suggests that long-lasting deficits in overall brain volumes persist into adulthood. Since lower brain volumes were correlated with lower IQ, these findings also have a long-lasting clinical relevance.

This analysis of a large pooled cohort is one of the first studies to demonstrate long-term brain volumetric alterations in CHD, spanning from childhood to young adulthood. Although our cohort has more children or adolescents (under 18) than adults (over 18), and is not extensive enough to cover adults over 32, given that there are no drastic changes in most of adulthood (before late adulthood), our findings enrich the existing literature; while numerous studies have documented reduced brain volumes at various ages in CHD (Bolduc et al., 2018; Meuwly et al., 2019; Heye et al., 2018; Melazzini et al., 2019; Peyvandi et al., 2019; Claessens et al., 2016; Naef et al., 2021; Verrall et al., 2021; von Rhein et al., 2015; Neukomm et al., 2022; Lee et al., 2024a), none have investigated age-related effects throughout adolescence into adulthood, during which extensive brain maturational processes occur.

### 4.1 Differences in grey and white matter development

We found a reduction not only in total brain volume but also in WM and GM volumes in the CHD group at any age, as has been previously reported (Watson et al., 2016; Easson et al., 2020). It should also be noted that our significant associations between CHD, age, or sex and the brain volumetric measures were strong enough to easily withstand a Bonferroni correction (obtained by multiplying the p-values by 4) to account for multiple tests.

While global brain volumes are valuable measurements for neurodevelopmental studies, it is also crucial to investigate specific tissue subtypes of the brain (WM, GM) separately because they develop and mature at different growth rates and trajectories (Gilmore et al., 2018). Measuring both GM and WM development is particularly important for CHD populations as the underlying biological mechanism for the reduction in WM and GM volume could be different. For example, WM maturity can be hindered when pre-myelinating oligodendrocyte progenitor cells are damaged due to hypoxia-ischemia during gestation, which can eventually impact white matter tract density (Back et al., 2001; Back and Miller, 2014). On the other hand, cortical grey matter expansion might be restricted in CHD due to the reduction in neuroblast numbers in the subventricular zone and alterations in interneuron and excitatory neuronal cell populations in the cortex (Morton et al., 2017).

In our pooled cohorts of CHD and controls, ages 9 to 32, we identified different brain size slopes in GM and WM, with the WM showing an overall increase and in contrast, the GM showing a decrease linearly with age. Our results are in line with previous research into brain maturation patterns in healthy populations; the WM volume linearly increases with age from birth to adulthood, reaching a plateau later in life, while the GM volume growth curve is somewhat close to an inverted U shape with a peak just before adolescence, decreasing thereafter (Lebel et al., 2012; Tamnes et al., 2010; Lebel and Beaulieu, 2011; Aubert-Broche et al., 2013). Our dataset did not allow us to evaluate a potential GM peak before adolescence, as we had only a few participants aged 9–10 years and no younger cases.

### 4.2 Comparison with other risk populations

Even though there is only scarce literature on GM and WM growth trajectory in the CHD population beyond infancy, our results might be comparable to studies on the preterm-born populations because both preterm-born and CHD populations share common neuropathology due to hypoxia-ischemic injury to the white matter from brain immaturity (Licht et al., 2009). Preterm-born populations are also reported to suffer from cognitive impairment (e.g., lower IQ, executive function) related to brain abnormalities (Omizzolo et al., 2014; Nosarti et al., 2014; Inder et al., 2023) similar to those seen in CHD populations (Easson et al., 2023). A recent longitudinal study of the very preterm-born population from birth to adolescence (measured at 0, 7, and 13 years old) showed that children born very preterm displayed increasingly reduced cortical volumes, particularly during ages 0–7 years, and by the age of 13, their cortical volumes had almost plateaued and were persistently lower than the term-born group (Kelly et al., 2024), in line with previous preterm studies (Monson et al., 2016; Thompson et al., 2020; Sripada et al., 2018). One preterm study with a meta-analytic approach found that preterm-born individuals followed the same GM and WM growth trajectories as term-born individuals, but volumes were consistently lower in preterm-born individuals from infancy to early adulthood with no evidence for catch-up growth (Schmitz-Koep et al., 2021). Our findings in brain development and maturation slope in CHD and controls are similar to these previous reports in preterm populations, indicating the persisting impact of early brain immaturity on long-term brain development.

### 4.3 Types of CHD and brain development

We found more pronounced brain volumetric alterations in patients with univentricular heart defects than in those with biventricular heart defects, and in patients with cyanotic defects than in those with acyanotic defects, in line with previous studies (Bolduc et al., 2018). This is not surprising, as the types and severity of CHD affect patients' brain development and growth trajectories due to different cardiovascular circulation, resulting in altered brain perfusion and oxygenation (Ibuki et al., 2012; Watanabe et al., 2009). For example, fetal MRI studies indicate slower brain growth, particularly affecting fetuses with HLHS and TGA (Rollins et al., 2021; Limperopoulos et al., 2010; Clouchoux et al., 2013; Jørgensen et al., 2018). Between 18 and 40 weeks of gestation, longitudinal brain growth was slower in HLHS and TGA but was similar in other CHD compared to controls, indicating an impact of the cardiac defect type on brain growth (Rollins et al., 2021). In one study, perioperative brain growth was slower in neonates with univentricular CHD compared to neonates with TGA, possibly related to differences in postoperative cardiac physiology (Peyvandi et al., 2018).

Although we had a variety of CHD diagnoses in our cohort, we did not have sufficient power to investigate differences in the brain size slope for different heart defects, especially for those with univentricular CHD. Our sensitivity analysis yielded broadly similar results using the same linear mixed model for both cyanotic and acyanotic CHD compared to the brain size slopes from a whole CHD sample, but the TBV, WM, and GM volume from the cyanotic CHD group remained lower and CSF slightly higher than those from the acyanotic CHD group. Previous studies in adults with univentricular CHD indicate persisting deficits in brain volume and brain growth, which should be further investigated in this vulnerable patient population as well (Verrall et al., 2021; Cordina et al., 2014).

### 4.4 CSF volumes in CHD populations

Despite smaller WM and GM volumes in CHD, there was no significant difference in the CSF volume in CHD and controls. This finding contrasts previous findings of studies showing larger CSF volume in CHD than controls (Owen et al., 2011; Heye et al., 2018; Cordina et al., 2014; Brossard-Racine et al., 2014). However, among our CHD group, univentricular CHD or those who underwent multiple surgeries showed larger CSF volumes than biventricular CHD or those with only one surgery, suggesting the complexity of the CHD contributes to the CSF enlargement, in line with previous studies (Heye et al., 2018; Rajagopalan et al., 2018). Although the mechanism of CSF alterations is not entirely clear, possible pathophysiology could be explained by delayed parenchymal brain development (Brossard-Racine et al., 2014), disrupted CSF absorption due to increased cerebral venous pressure (Puntis et al., 2016) and abnormal ciliary function (Panigrahy et al., 2016). Even though our results did not show statistically significant differences between CHD and controls, our findings still imply that patients with CHD had a higher CSF/brain volume ratio, with smaller total brain volume and similar CSF volume compared to controls beyond childhood and adolescence, suggesting the long-term CSF alterations. Our study did not specifically assess extra-axial CSF, as we combined ventricular and non-ventricular CSF to define global CSF volume, but some studies have reported enlargement of extra-axial CSF, especially in fetal and neonatal populations (Brossard-Racine et al., 2014; Schellen et al., 2015; Mlczoch et al., 2013; Kelly et al., 2017; Ng et al., 2020). Precise assessment and tracking of extra-axial CSF would be an interesting avenue for future research.

Our study did not find a statistically significant relationship between CSF and IQ; however, some reported the connection between altered CSF and cognitive functions in neonates (Heye et al., 2018; Knirsch et al., 2017) as well as in adolescents and young adults (Lee et al., 2024b). This could be attributed to the complexity of CHD in our cohort, as our cohort included many mild and moderate CHD cases. Since the literature on CSF alterations in CHD beyond childhood remains scarce (Cordina et al., 2014), it is crucial to track adult CHD populations, including complex CHD cases, given the possible connection with cognitive functions.

### 4.5 Clinical implications

We confirmed the association between smaller TBV, WM, and GM volumes and associations with lower IQ, which is in line with previous studies (Bolduc et al., 2018; Brossard-Racine and Panigrahy, 2023). Childhood and adolescence represent a critical period in extensive neural maturation (Gilmore et al., 2018; Tamnes et al., 2010; Lebel et al., 2008), as well as the period when neurodevelopmental problems tend to become more apparent for patients with CHD as a higher level of function is required (Latal, 2016; Feldmann et al., 2021). Thus, our findings of age-related changes in brain size slope in CHD and its consistent association with cognitive function highlight the importance of monitoring brain growth in this population. In addition, we confirmed that low maternal education was associated with smaller TBV, WM and GM volumes. The functional implications of smaller brain volumes are more pronounced, especially in patients with CHD from low socioeconomic backgrounds.

### 4.6 Limitations

There are several limitations to this study. First and foremost, we analysed a collection of cross-sectional data with statistical models and did not track the brain growth trajectories longitudinally. The true brain developmental trajectories in CHD can be tracked only through longitudinal follow-up studies. This collection of different samples also contributed to the difference in IQ across ages (i.e., the oldest sample had a relatively lower IQ than the younger samples; this age-IQ association, although minimal, was not expected). We have to note that, three cohorts used a short-form IQ (which was corrected to reduce bias), and the oldest cohort used a full-form IQ, which might have introduced measurement variability. Also, as we pooled data from different time periods, the era during which subjects underwent the first surgery varied across cohorts. However, including a random study effect in all models helps account for systematic differences between cohorts, including the fact that surgeries occurred in different eras and with different clinical practices. Despite our cross-sectional design with limited age groups, we were able to include a large number of patients, which would have been difficult to maintain in longitudinal studies. Furthermore, we replicated the brain size slopes in TBV, WM, GM and CSF from 9 to 32 years old, observing a similar age effect to that reported in previous longitudinal studies of normative populations (Lebel et al., 2012; Lebel and Beaulieu, 2011; Aubert-Broche et al., 2013). However, the GM size slope could have been better replicated with younger children (e.g., under 9 years old, for which we did not have data), as studies show an inverted U-curve with its peak just prior to adolescence (Giedd et al., 1999). Nonetheless, the decrease in GM in our sample was similarly described in other studies in healthy participants (Lebel et al., 2012). It is also noteworthy that we conducted our analysis with Freesurfer version 5.3, thus, our specific brain volume values might not be directly comparable to those from studies conducted with more recent versions (Bigler et al., 2020; Filip et al., 2022). Another limitation is that there were some significant differences in maternal education between patients with CHD and controls (controls had significantly higher maternal education). While we included maternal education in our models, residual confounding is still possible given the broad effects of maternal education on development. Moreover, a single-centre Swiss study that excludes non-German speakers limited its external validity. Furthermore, the final sample was older and underrepresented individuals with cyanotic or univentricular CHD, reflecting selective attrition common in MRI studies. Although exclusions were mainly due to MRI contraindications or image quality (e.g., motion artefacts), this may limit generalizability to more severe CHD. Additionally, we could not consider the impact of puberty on brain development in our dataset, although puberty influences brain structural development (Blakemore et al., 2010; Vijayakumar et al., 2018), and there might be a group difference in the timing of puberty between healthy and CHD populations (Haberer and Silversides, 2019). Although we have captured the global effects of CHD, which was our main purpose of this study, region-specific volumetric analyses (e.g., hippocampus, cerebellum) would have provided additional insight beyond global measures and should be addressed in future work. Lastly, we were not able to capture the potential differences in brain size slopes of specific types of CHD (those slopes were depicted with the same models) as our sample included a variety of CHD diagnoses. In particular, there were only a few patients with univentricular CHD among the older participants, making it difficult to evaluate the impact of uni-vs biventricular CHD on brain size slopes across the full age range of the cohort.

## Funding

This project was supported by the 10.13039/100002129Swiss Heart Foundation, the Swiss National Science Foundation, the Mäxi Foundation, the Else Kröner-Fresenius Foundation, 10.13039/501100008477Heuberg Stiftung, 10.13039/501100014357Neuroscience Center Zurich and the Olga-Mayenfisch Foundation.

## CRediT authorship contribution statement

**Asuka Toyofuku:** Formal analysis, Investigation, Methodology, Project administration, Software, Writing – original draft, Writing – review & editing. **Nadja Naef:** Conceptualization, Data curation, Writing – original draft, Writing – review & editing. **Valentin Rousson:** Formal analysis, Investigation, Visualization, Writing – review & editing. **Melanie Ehrler:** Conceptualization, Data curation, Methodology, Writing – review & editing. **Flavia M. Wehrle:** Data curation, Funding acquisition, Writing – review & editing. **Ladina Schlosser:** Data curation, Methodology, Writing – review & editing. **Michael von Rhein:** Data curation, Funding acquisition, Writing – review & editing. **Rabia Liamlahi:** Data curation, Writing – review & editing. **Matthias Greutmann:** Data curation, Methodology, Writing – review & editing. **Oliver Kretschmar:** Methodology, Writing – review & editing. **Beatrice Latal:** Conceptualization, Funding acquisition, Supervision, Writing – review & editing. **Ruth Tuura O'Gorman:** Funding acquisition, Investigation, Methodology, Supervision, Writing – review & editing.

## Declaration of competing interest

The authors declare the following financial interests/personal relationships which may be considered as potential competing interests:We declare that we have no competing interests.

## Data Availability

Data will be made available on request.
