# Supplementary material for: Brain volumes in congenital heart disease from childhood to adulthood: A pooled neuroimaging study
Source: Neuroimage Rep. 2026 Jul 8;6(3):100379. doi: 10.1016/j.ynirp.2026.100379 (PMC13380037; doi:10.1016/j.ynirp.2026.100379)
Supplement: Multimedia component 1 [file mmc1.docx]

**Supplemental Figure 1: Flowchart**

**
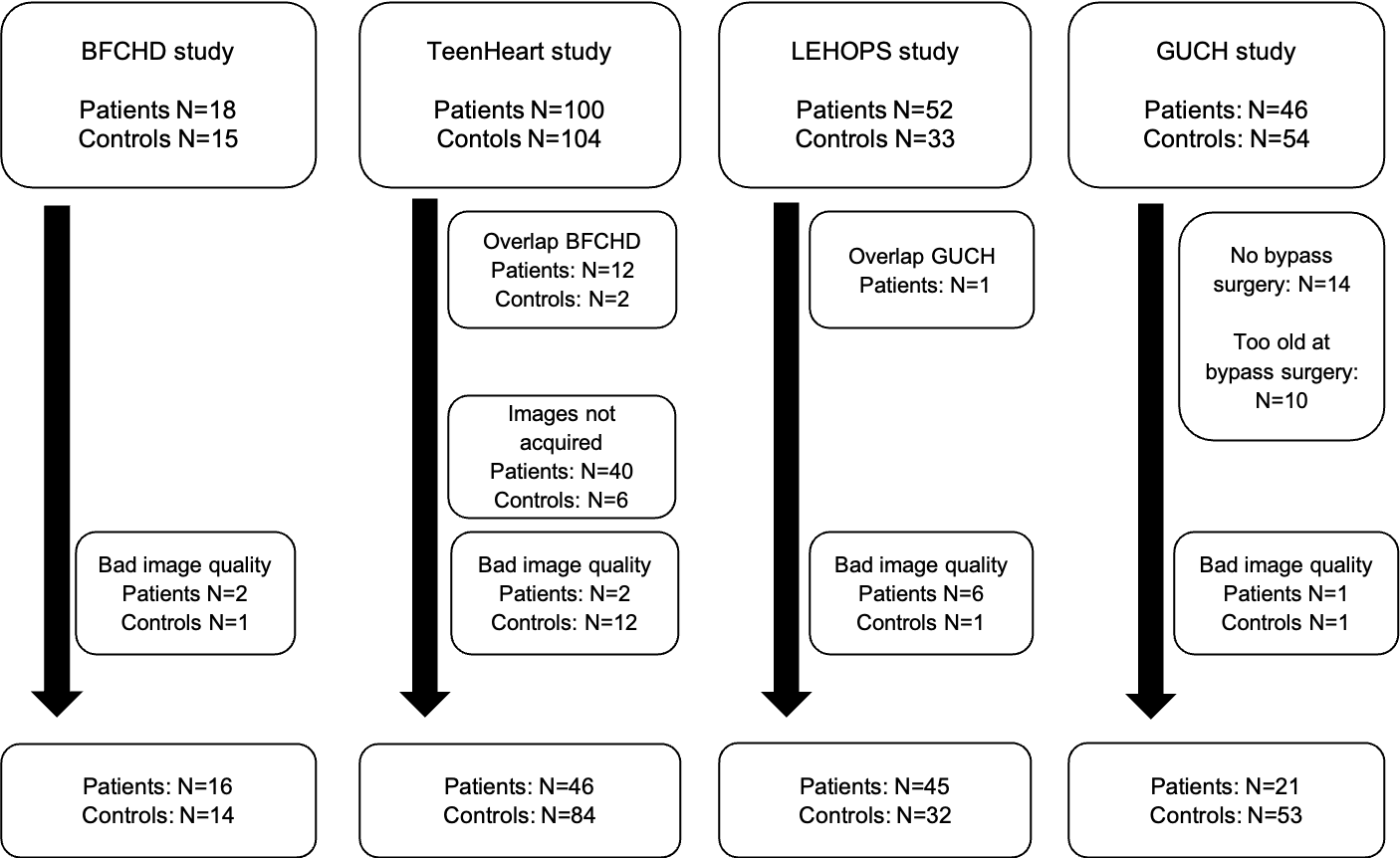
**

**Supplemental Table 1: Characteristics of CHD groups**

|  | **Univentricular (N= 14)** | **Biventricular**  **(N= 114)** | **p-value** | **Cyanotic**  **(N= 74)** | **Acyanotic**  **(N= 54)** | **p-value** |
| --- | --- | --- | --- | --- | --- | --- |
| Male, N (%) | 9 ( 64.3) | 66 ( 57.9) | 0.864 | 47 ( 63.5) | 28 ( 51.9) | 0.254 |
| Age(year), Mean (SD) | 14.72 (5.14) | 15.45 (5.55) | 0.642 | 14.65 (5.52) | 16.35 (5.34) | 0.082 |
| Maternal education, Mean (SD) | 3.64 (1.15) | 3.82 (1.21) | 0.596 | 3.79 (1.19) | 3.82 (1.21) | 0.875 |
| IQ, Mean (SD) | 93.24 (9.87) | 102.08 (13.14) | 0.017 | 99.68 (13.03) | 103.11 (13.02) | 0.147 |
| Age 1st bypass surgery,  months Median [IQR] | 3.72 [0.27, 6.92] | 4.46 [0.47, 10.63] | 0.327 | 0.99 [0.24, 7.41] | 7.08 [3.43, 27.36] | <0.001 |
| One bypass surgery, N (%) | 2 ( 14.3) | 93 ( 81.6) | <0.001 | 52 ( 70.3) | 43 ( 79.6) | 0.460 |

Mean (SD), N (%), Median [IQR], IQ: Intelligence quotient, CHD: congenital heart disease

**Supplemental Table 2: CHD diagnosis per cohort**

| Diagnosis | BFCHD | Teen | LEHOP | GUCH | whole |
| --- | --- | --- | --- | --- | --- |
| d-Transposition of the Great Arteries (d-TGA) | 7 | 12 | 10 | 6 | 35 |
| Ventricular Septal Defect (VSD) | 0 | 10 | 13 | 6 | 29 |
| Tetralogy of Fallot (TOF) | 1 | 5 | 6 | 3 | 15 |
| Coarctation of the Aorta (CoA) | 1 | 1 | 1 | 2 | 5 |
| Hypoplastic Left Heart Syndrome (HLHS) | 1 | 4 | 0 | 0 | 5 |
| Total Anomalous Pulmonary Venous Connection (TAPVC) | 2 | 1 | 2 | 0 | 5 |
| Atrioventricular Canal Defect (AVCD) | 2 | 2 | 0 | 0 | 4 |
| Aortic Stenosis (AS) | 0 | 0 | 4 | 1 | 5 |
| Pulmonary Atresia (PA) | 0 | 2 | 1 | 0 | 3 |
| Other Functional Single Ventricle Anomaly | 0 | 1 | 0 | 0 | 1 |
| Pulmonary Stenosis (PS) | 0 | 0 | 2 | 0 | 2 |
| Truncus Arteriosus | 0 | 2 | 0 | 0 | 2 |
| Atrial Septal Defect (ASD) | 0 | 0 | 1 | 1 | 2 |
| Mitral Valve Disease with ASD | 0 | 1 | 0 | 0 | 1 |
| Mitral Valve Disease with VSD | 0 | 0 | 0 | 1 | 1 |
| Isolated Congenital Aortic Valve Disease | 0 | 0 | 0 | 1 | 1 |
| Patent Ductus Arteriosus (PDA) | 0 | 0 | 1 | 0 | 1 |
| Shone Complex | 0 | 0 | 1 | 0 | 1 |
| Tricuspid Atresia (TA) | 0 | 0 | 1 | 0 | 1 |
| Double Inlet Left Ventricle (DILV) | 0 | 0 | 1 | 0 | 1 |
| Other combined diagnosis* | 2 | 5 | 1 | 0 | 8 |

*Other cases example: Abnormal origin of the left pulmonary artery from the descending thoracic aorta and VSD

**Supplemental Table 3: Brain volumes between 9-32 years old, sensitivity analysis**

|  |  | Effect | delta | 95% CI | p-values |  | Effect | delta | 95% CI | p-values |
| --- | --- | --- | --- | --- | --- | --- | --- | --- | --- | --- |
| **only biventricular CHD** | **TBV** | group CHD | −0.77 | −1.03;−0.51 | <0.001 | **WMV** | group CHD | −0.75 | −1.01;−0.49 | <0.001 |
|  |  | sex male | 1.08 | 0.84;1.31 | <0.001 |  | sex male | 0.88 | 0.64;1.12 | <0.001 |
|  |  | age | −0.18 | −0.42;0.06 | 0.14 |  | age | 0.3 | 0.07;0.52 | 0.009 |
|  |  | maternal education | 0.22 | 0.1;0.35 | <0.001 |  | maternal education | 0.2 | 0.07;0.32 | 0.002 |
|  | **GMV** | group CHD | −0.67 | −0.93;−0.42 | <0.001 | **CSF** | group CHD | 0.01 | −0.24;0.26 | 0.95 |
|  |  | sex male | 1.12 | 0.89;1.36 | <0.001 |  | sex male | 0.33 | 0.09;0.56 | 0.006 |
|  |  | age | −0.58 | −0.82;−0.34 | <0.001 |  | age | 0.26 | 0.14;0.38 | <0.001 |
|  |  | maternal education | 0.22 | 0.09;0.34 | <0.001 |  | maternal education | 0.11 | −0.01;0.23 | 0.075 |
|  |  | Effect |  |  |  |  | Effect |  |  |  |
| **only cyanotic CHD** | **TBV** | group CHD | −1 | -1.3;-0.71 | <0.001 | **WMV** | group CHD | -1.00 | -1.3;-0.71 | <0.001 |
|  |  | sex male | 1.02 | 0.76;1.27 | <0.001 |  | sex male | 0.82 | 0.56;1.07 | <0.001 |
|  |  | age | −0.18 | -0.42;0.06 | 0.15 |  | age | 0.32 | 0.14;0.51 | <0.001 |
|  |  | maternal education | 0.24 | 0.1;0.38 | <0.001 |  | maternal education | 0.2 | 0.06;0.33 | 0.005 |
|  | **GMV** | group CHD | −0.87 | -1.17;-0.58 | <0.001 | **CSF** | group CHD | 0.26 | -0.04;0.55 | 0.088 |
|  |  | sex male | 1.08 | 0.83;1.34 | <0.001 |  | sex male | 0.27 | 0.01;0.52 | 0.038 |
|  |  | age | −0.57 | -0.82;-0.32 | <0.001 |  | age | 0.27 | 0.12;0.43 | <0.001 |
|  |  | maternal education | 0.26 | 0.12;0.4 | <0.001 |  | maternal education | 0.07 | -0.07;0.2 | 0.34 |
|  |  | Effect |  |  |  |  | Effect |  |  |  |
| **only acyanotic CHD** | **TBV** | group CHD | -0.67 | -1;-0.34 | <0.001 | **WMV** | group CHD | -0.66 | -0.99;-0.33 | <0.001 |
|  |  | sex male | 1.13 | 0.87;1.39 | <0.001 |  | sex male | 0.94 | 0.68;1.2 | <0.001 |
|  |  | age | -0.16 | -0.43;0.12 | 0.26 |  | age | 0.32 | 0.07;0.57 | 0.013 |
|  |  | maternal education | 0.18 | 0.03;0.32 | 0.015 |  | maternal education | 0.15 | 0;0.29 | 0.044 |
|  | **GMV** | group CHD | -0.56 | -0.89;-0.23 | <0.001 | **CSF** | group CHD | -0.05 | -0.37;0.28 | 0.78 |
|  |  | sex male | 1.15 | 0.89;1.41 | <0.001 |  | sex male | 0.44 | 0.18;0.7 | 0.001 |
|  |  | age | -0.56 | -0.82;-0.3 | <0.001 |  | age | 0.25 | 0.12;0.38 | <0.001 |
|  |  | maternal education | 0.18 | 0.03;0.32 | 0.015 |  | maternal education | 0.06 | -0.08;0.21 | 0.37 |

TBV: Total brain volume, WM: Total white matter, GM: Total grey matter, CSF: Cerebrospinal fluid, CI = confidence interval


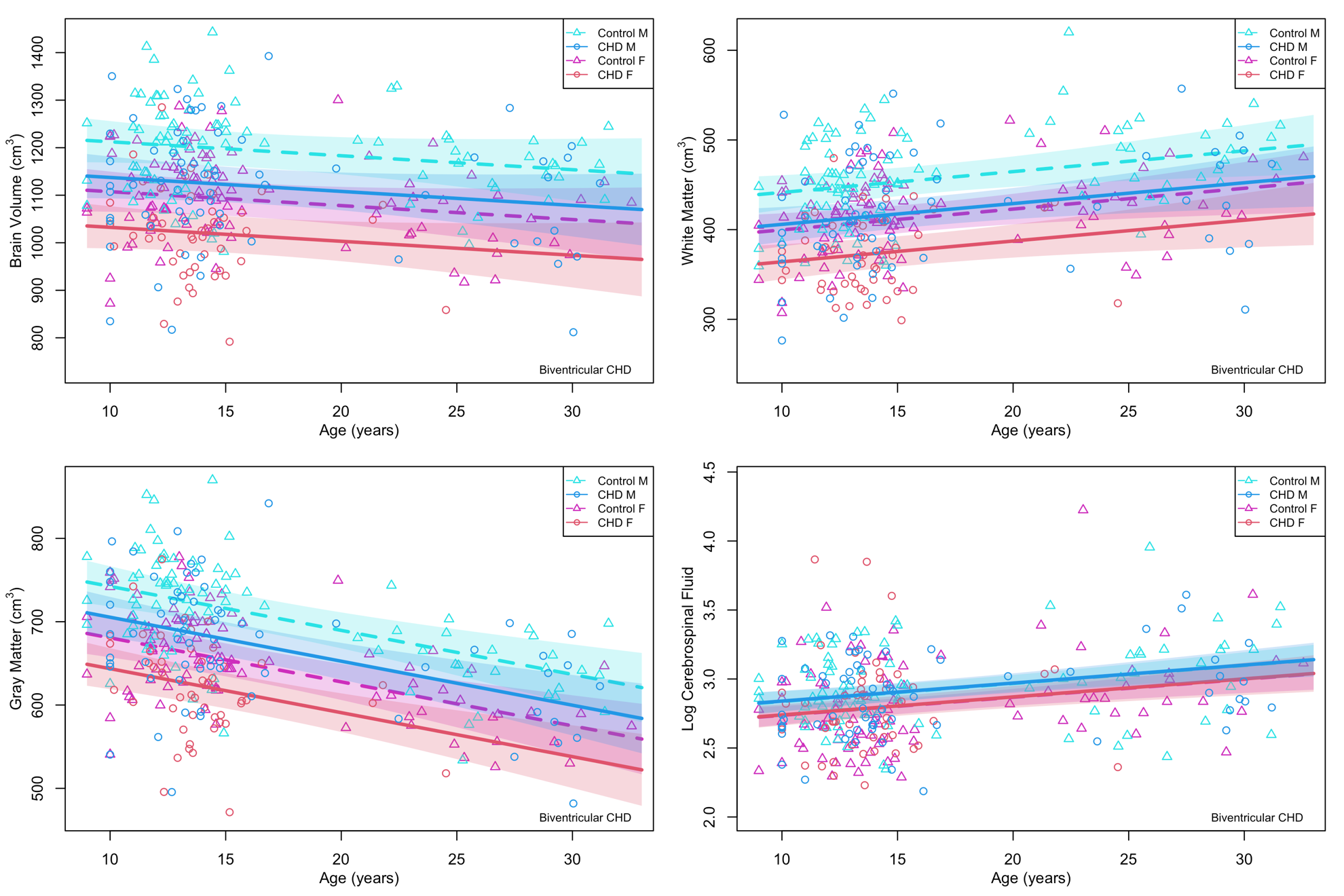


**Supplemental Figure 2: brain curves from childhood to adulthood in controls and patients with biventricular CHD**

For the sensitivity analysis, the curves were plotted excluding those with univentricular CHD


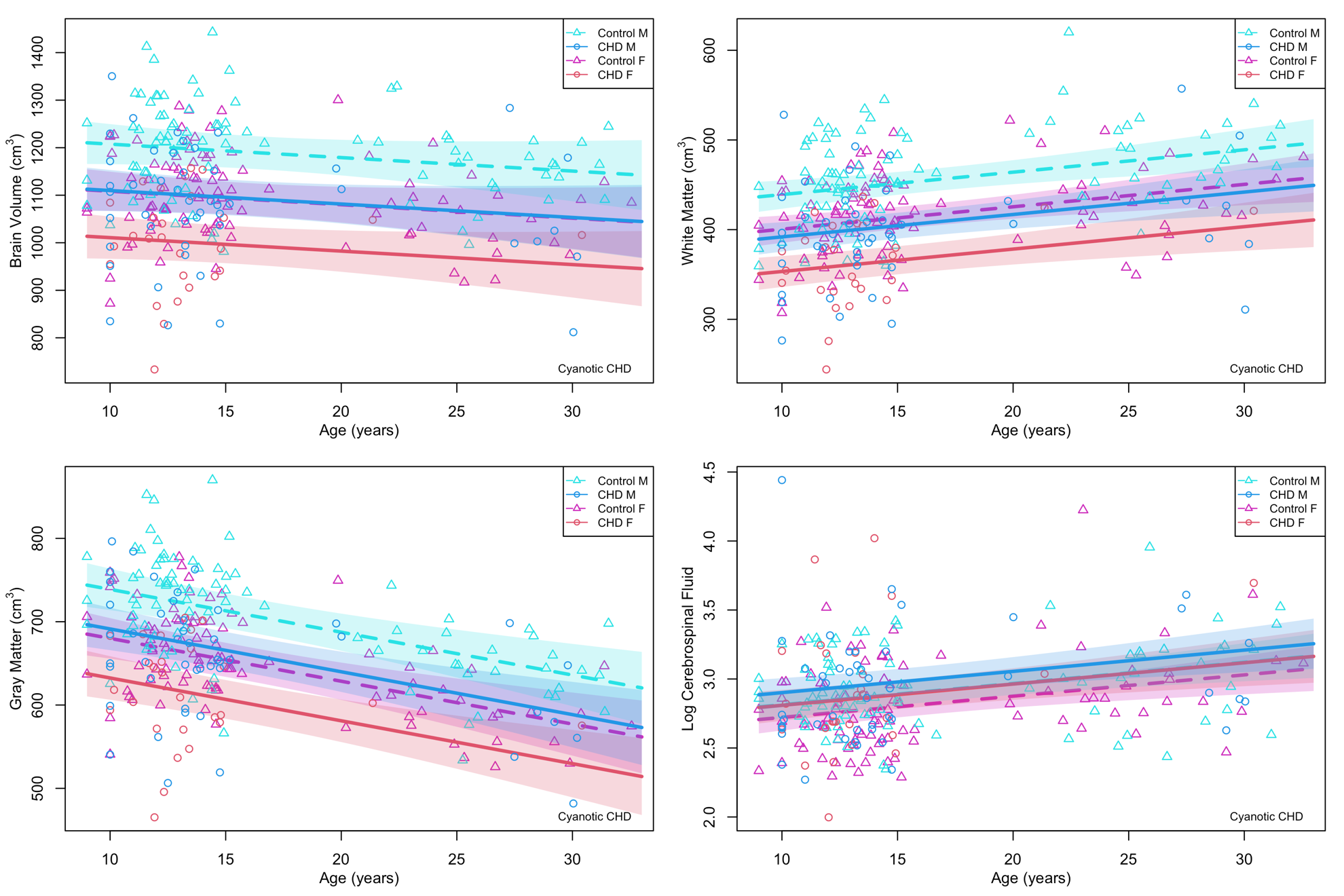


**Supplemental Figure 3: brain curves from childhood to adulthood in controls and patients with cyanotic CHD**

For the sensitivity analysis, the curves were plotted excluding those with acyanotic CHD.


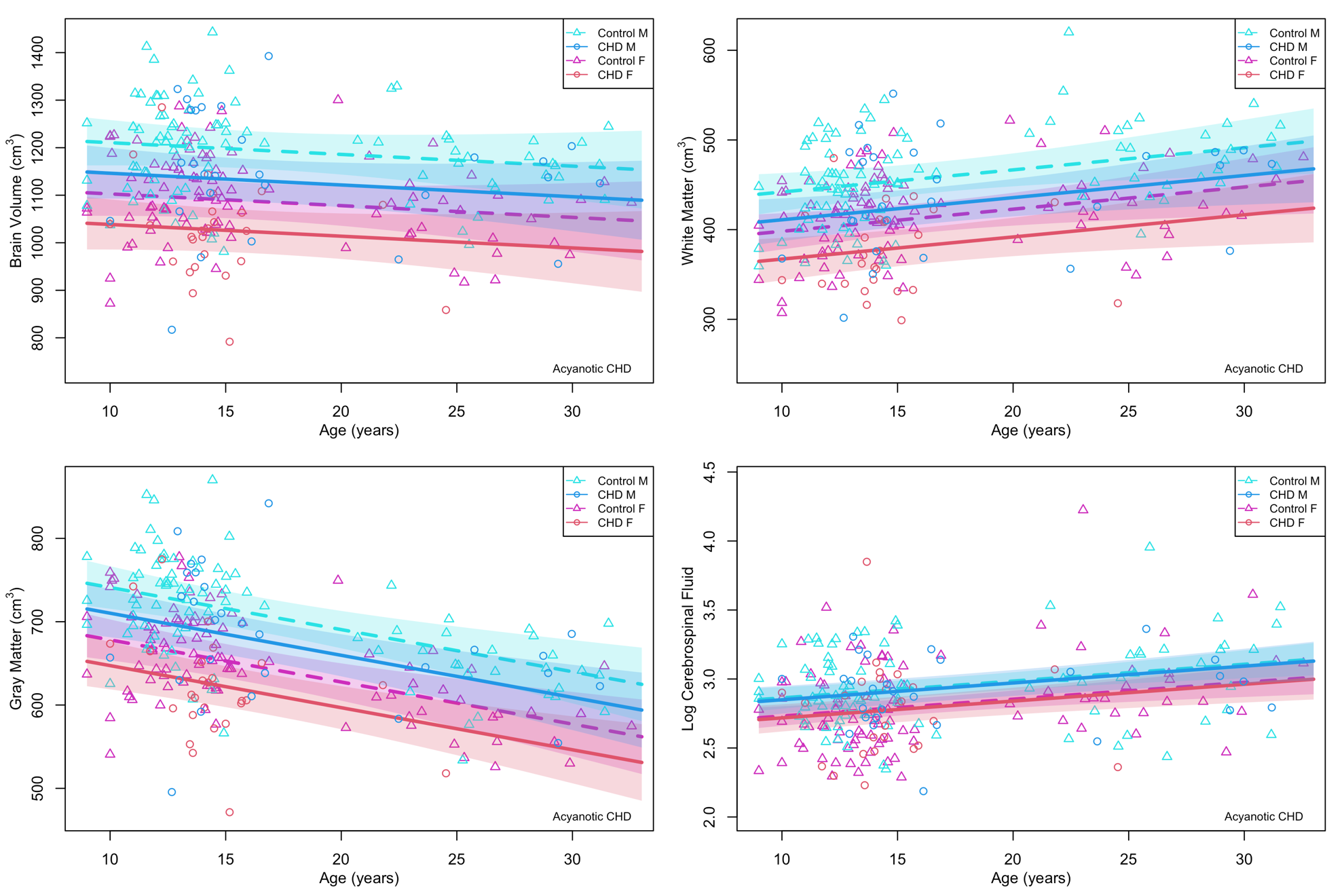


**Supplemental Figure 4: brain curves from childhood to adulthood in controls and patients with acyanotic CHD**

For the sensitivity analysis, the curves were plotted excluding those with cyanotic CHD.
